# Challenges for ATM management in times of market variability caused by the COVID-19 pandemic crisi

**DOI:** 10.1007/s10100-022-00816-2

**Published:** 2022-11-10

**Authors:** Marcin Suder, Tomasz Wójtowicz, Rafał Kusa, Henryk Gurgul

**Affiliations:** Faculty of Management, AGH University of Science and Technology, ul. Gramatyka 10, 30-067 Krakow, Poland

**Keywords:** Automatic teller machine (ATM), Crisis management, COVID-19, Event study analysis

## Abstract

One of the most important issues related to the management of an Automatic Teller Machine (ATM) network is the correct forecasting of the demand for cash. Typically, this demand, expressed as the value or number of ATM withdrawals, has some regularities that can be used to evaluate future values for these variables. However, forecasting becomes a challenge when a crisis occurs that could affect the behavior of ATM users. In this context, it is important to identify how the development of the crisis and the various information concerning it may affect people’s attitudes to cash.

This study aims to examine the impact of the COVID-19 pandemic on the behavior of ATM customers. On the basis of daily data from 81 ATMs, we analyze the changes in the value and number of withdrawals just before and during the COVID-19 pandemic in Poland. An event study analysis allows us to determine precisely the moments in which changes in user behavior took place. This means that it is also possible to examine the reaction of ATM users to the announcement and implementation of the pandemic restrictions, and to determine the factors that had an impact on the change in people’s attitude to cash. Such a study is also important from a sociological point of view, as it enables one to understand people’s reactions to the emerging crisis. Hence, its results may be useful not only for managers of ATM networks, but also for various authorities and policy makers.

## Introduction

The process of Automatic Teller Machine (ATM) network management, and especially cash management in ATM networks, is important both for the network operator and for customers using ATMs. For operators, it is related to profit optimization, and for customers, it is related to access to cash. Managing an ATM network is a multi-stage process that requires the manager to act on many levels and to cooperate with many institutions. Incorrect decisions related to network management (which include the selection of a location for an ATM, the amount of time required to top up, and the logistics of transporting cash, among other things) may result in a significant increase in service costs. The main elements of cash service costs, which range from 35 to 50%, are the costs of transporting cash between cash points and the costs of lost opportunities (alternative use of cash) as a result of money being frozen (in the case of this latter cost, the interbank rate is most often assumed). Therefore, one of the basic tasks in the ATM network management process is to minimize the global costs of handling cash, while ensuring a sufficiently high and stable level of cash availability (Gurgul and Suder 2016). However, these are opposing and at the same time inseparable goals of the cash management process, which is why the topic of savings is intertwined here with the issue of availability. In the case of an ATM, the unavailability of money implies a number of unfavorable phenomena for the bank or network operator. Above all, it causes an increase in customer dissatisfaction and the loss of their trust, which needs to be avoided.

The information presented above regarding the costs of operating an ATM network and the related cash management indicate the need for an improvement in the methods employed in forecasting the number and value of ATM withdrawals. A correct forecast of the size of withdrawals can noticeably reduce the costs associated primarily with “freezing” cash, improving the transport of cash to an ATM, or reducing the quantity of cash returns that remain unused in the ATM. However, a prerequisite for making an appropriate forecast (with the smallest error) is to know the past behavior of customers in terms of the amounts and number of ATM withdrawals. This is usually achieved through a detailed analysis of the time series that describes the number of payouts and their size on a daily basis. The possibility of using historical data is limited by the assumption that the environmental conditions in which both managers and clients operate do not change. The onset of the COVID-19 pandemic and the resulting restrictions dramatically changed these conditions. In particular, there was panic and concern among the public about the uncertainty the pandemic brought. In addition, various restrictions were imposed both unexpectedly and with immediate effect, increasing the feeling of insecurity. These circumstances greatly influenced the payment behavior of ATM customers. In a crisis situation, the skillful management of an ATM network requires an appropriate analysis of customer behavior, in particular their reaction to events that are usually unpredictable or predictable but only in the very short term. Such knowledge can noticeably improve the cash management process in an ATM network. A better understanding of consumer habits and behavior is essential when it comes to managing an ATM network, particularly in terms of the logistics of delivering cash to ATMs.

This research aims to identify changes in the behavior of ATM clients caused by the COVID-19 pandemic. On the basis of daily data from 81 ATMs installed in Krakow (the second largest city in Poland) we study changes in the value and number of withdrawals. The event study allows us to determine precisely the moment when significant changes in the behavior of ATM users occurred, resulting in e.g. an increase or decrease in the value of ATM withdrawals. The results obtained can also be compared to information on the development of the pandemic, in particular to the times of announcements and the introduction of various pandemic restrictions. This comparison makes it possible to identify the factors that changed people’s attitude to cash.

This study is intended to contribute to research into cash management in banking systems. In particular, it fills a gap in research on the structure of ATM withdrawals. While the ATM has remained one of the most important means of accessing cash in the last decade, literature on ATM operation, logistics, cash transportation and ATM cash management is relatively scarce. The paucity of publications dealing with the issue of cash management in ATM networks can be explained by researchers’ lack of access to data related to cash turnover in ATMs. This study also aims to provide ATM operators with an analysis of ATM management in times of lockdown. In particular, the results of the study may help ATM operators to predict changes in withdrawals resulting from a government’s announcements regarding restrictions it plans to introduce. This can serve as a basis for the decision-making process regarding ATM inventory management in times of high market volatility and uncertainty.

In the next part, the literature is reviewed. In the following section, the methods and data are described. Then, the results of the computations are presented and discussed. Finally, the conclusions are presented.

## Literature overview

Banks are subject to dynamic changes that arise from developments in technology, which prompt many banking systems to adopt demonetization (e.g., Sam et al. 2021). Cashless payments make it easier for users to perform financial transactions and enable businesses to save both time and the costs of cash management. Moreover, electronic payments create opportunities to expand business and increase sales. Consequently, banks adopt digital payment means as well as various mobile banking solutions. This process is supported both by policies on a national level and by banks themselves. Despite these changes, cash is still used for transactions. Due to financial and technological constraints, electronic payment methods may not be universally available in every country (Cevik 2020). Additionally, cash payment is still preferred by some groups of clients, for example by consumers for whom it is important to remain in control of their remaining liquidity and whose information processing costs are elevated (Alckreuth et al. 2014). Thus, even in developed countries, there is a large number of easily accessible ATMs. Moreover, in countries whose banking systems are still inadequate in terms of customer demand, an increase in the distribution of ATMs has been reported (Adane et al. 2021). However, ongoing demonetization results in a decreasing number of ATM transactions in the long term (Fouillet et al. 2021).

ATM operators strive to optimize inventory management and lower operation costs. This pertains to balancing ATM location distribution (Bilginol et al. 2015), routes, periods or quantities delivered (Larrain et al. 2017). Other challenges include forecasting, the replenishment of ATMs and the denomination mix. For example, optimizing the cash inventory level and the time between orders (Baker et al. 2013) as well as implementing a time-varying denomination mix allow a significant reduction in the operational costs of managing an ATM.

The emergence of the COVID-19 pandemic has dramatically changed consumer behavior in retail banking, which has changed the strategy of institutions operating on the banking market (banks, ATM operators). This has been reflected in academic studies that address the subject of the impact of COVID-19 on the banking system. However, it should be noted that these studies usually concern payment habits in general and changing trends, while issues related to ATMs are only one of the elements of these studies.

Wójcik and Ioannou (2020) comment on the actual and potential influence of the pandemic on financial markets, the financial sector and centers. They expected a lower rate of new financial regulation during the pandemic. They also predicted that firm-level consolidation would continue, and that there would be a further rise in business services related to finance. They forecasted acceleration in the use of new financial technologies. The authors claimed that regional and local financial centers would probably face greater problems than large international centers.

Baicu et al. (2020) examine how the COVID-19 crisis impacted on consumer behavior in retail banking in the Romanian banking sector. The results of their study indicate, inter alia, that the perception of the impact of the COVID-19 pandemic on consumer lifestyles had a direct and positive impact on people’s attitudes to online and mobile banking services, through other variables such as the safety of internet and mobile banking services. Cevik (2020) draws attention to the fact that the spread of infectious diseases reduces the demand for physical cash, and in the long run, transaction restrictions related to the pandemic may accelerate the adoption of digital technologies around the world.

Hoe (2020) notes that due to restrictions on direct social contact, cash use was limited during the pandemic crisis and consequently digital banks in Southeast Asia witnessed an increase in bank account registrations. Craven et al. (2020) point to the significant increase in online services and shopping in China and believes that these changes are irreversible.

A noticeable impact on the operation of ATMs during the COVID-19 pandemic resulted from the approach to the use of cash and related regulations implemented by the governments of individual countries. A review of the behavior of governments and central banks of selected countries is carried out by Auer et al. (2020). Their research shows that these behaviors were diverse and there was no single model of behavior in this area. Some countries, including Poland, encouraged the use of cashless payments. This was dictated by the belief that a cashless payment could limit the spread of the virus (Yakean 2020). In many countries that had this approach to cash, new digital means of payment were implemented during the pandemic (Mansour 2021), which intensified the move away from cash. In this context, Auer et al. (2020) point out that switching entirely to digital payments can have a negative impact on older consumers without bank accounts. Therefore, a balance should be struck between the availability of digital payments (non-cash operations) and cash-based payments.

In some countries, e.g. China, South Korea and Hungary, it was believed that the use of cash could result in the virus spreading more quickly, but they did not recommend giving up cash, but instead decided to sterilize banknotes and quarantine them. However, The Bundesbank and the Reserve Bank of South Africa followed a different approach: they encouraged confidence in cash, reporting that the risk of coronavirus transmission through banknotes was low.

In turn, Pop (2020) draws attention to another issue. He notes that the outbreak of the COVID-19 pandemic has resulted in significant difficulties in the management of financial institutions such as banks or independent ATM operators. According to Pop, the rapid pace of change caused by the pandemic is requiring these institutions to adopt flexible approaches and marketing strategies. Similar conclusions are reached by Alharthi et al. (2021), who study disruptions (such as pandemic crises resulting in lockdowns) that affect the functioning of banks and customer satisfaction with the Islamic banking system. Banks and ATM operators need to adapt their business models to the societal changes associated with the COVID-19 crisis (PwC, 2020). In particular, due to changes in withdrawal habits, in the case of ATM operators they are forced to analyze the time series of withdrawals in detail and look for new models and methods of forecasting withdrawals that will be effective under market conditions similar to those caused by the COVID-19 pandemic.

This issue is dealt with by Fallahtafti et al. (2022). On the basis of data from Singapore, they attempt to forecast ATM cash demand for specific periods both before and during the COVID-19 pandemic. They provide proof that sophisticated models will not always perform better than simpler models. They find that during the COVID-19 pandemic and at times when there is a sudden increase in demand and massive volatility in the pattern of withdrawals, the statistical models of autoregressive integrated moving average (ARIMA) and seasonal ARIMA (SARIMA) are generally more effective in forecasting.

## Data and methods

The COVID-19 pandemic has dramatically affected economies all around the world. The restrictions have resulted in changes on both the supply and demand side. The lockdowns that were introduced have also affected people’s daily lives and their social interactions with others. Furthermore, the pandemic has changed people’s habits regarding e.g. work and shopping. This, in turn, has had an effect on people’s attitude to cash. These changes are of great importance from the point of view of managing the ATM network, particularly from the perspective of inventory management.

An important element of the research presented in this paper is the selection of those events related to the COVID-19 in Poland that may have had a significant impact on the behavior of ATM customers. Hence, it is worth first briefly describing the development of the COVID-19 pandemic in Poland, paying particular attention to events and information that could have had an impact on the behavior of ATM users. At the beginning of 2020, information about the increasing number of cases of SARS-CoV-2 in various parts of the world began to reach Poland. The development of the pandemic was particularly dramatic in Italy. However, Poland did not record its first laboratory confirmed case of SARS-CoV-2 until March 4, 2020. A few days later, on March 10 (Tuesday), the cancelation of mass events was announced. This was the first important pandemic restriction in Poland. The next day, another set of restrictions was announced. The most important were the closure of educational institutions (such as schools and universities)^1^ as well as that of various cultural institutions (such as museums and theaters). All these restrictions started on March 12 (Thursday). Subsequently, various other restrictions were introduced. In the context of the analysis presented in the paper it is also worth mentioning that in Poland the second wave of the COVID-19 pandemic occurred in the fall of 2020, while the third wave took place between February and April 2021. Mass vaccination against the virus started at the beginning of 2021, but in the first quarter of 2021 the number of people vaccinated was fairly limited.

**Fig. 1:**
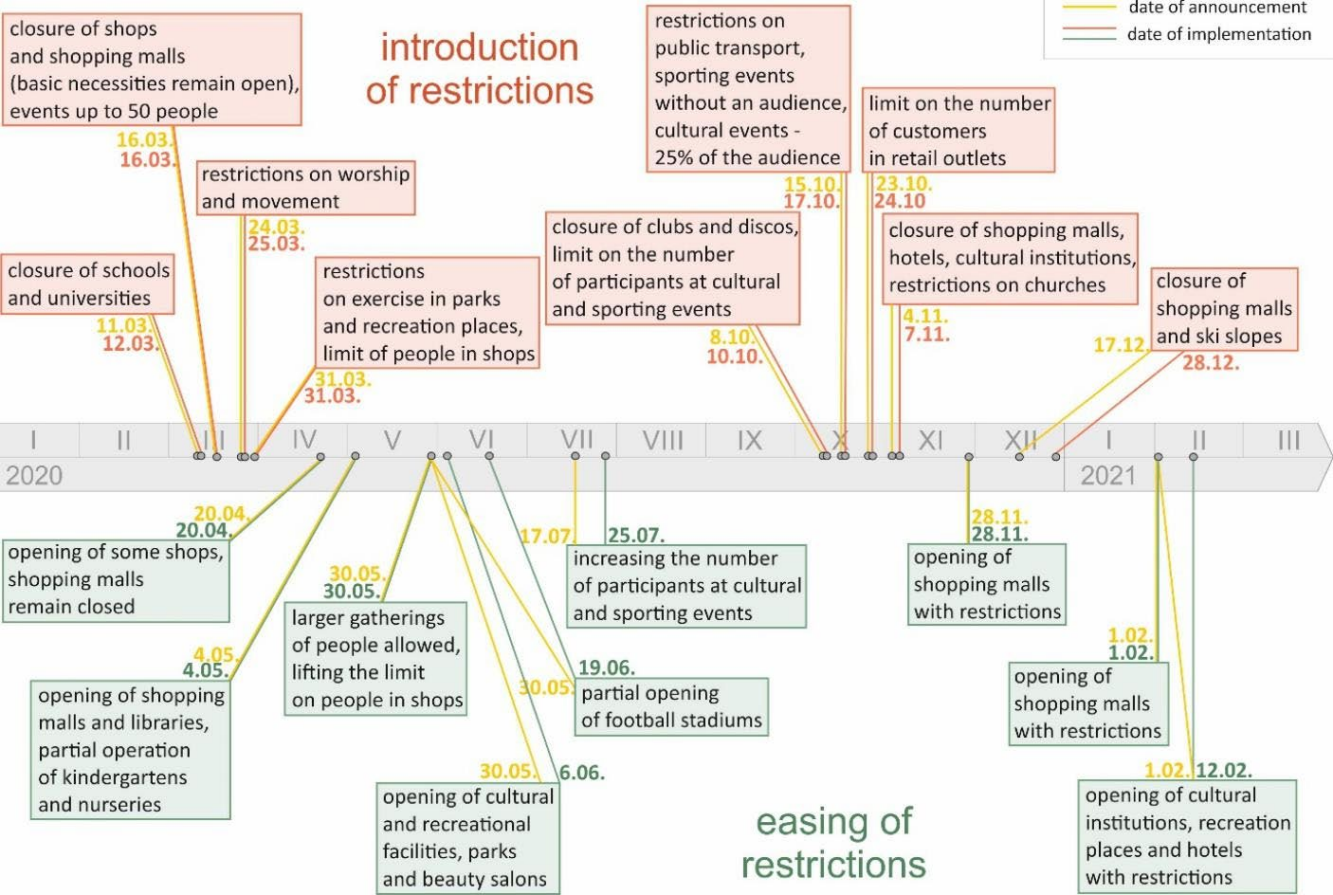
COVID-19 pandemic restrictions in Poland in 2020. Notes: In this Figure we present the most important announcements about the imposition (above the timeline) and lifting (below the timeline) of restrictions in Poland in 2020. For each restriction we report two dates: the date of the announcement of the new regulation and the date of its implementation

After a detailed analysis of all the announcements and legal regulations introduced by the government, we selected those that in our opinion could have directly affected the behavior of ATM customers. We took into account both the government’s decisions related to the introduction of restrictions and those related to lifting them. The dates on which these events were announced and when they were introduced are summarized in Fig. 1.

**Fig. 2:**
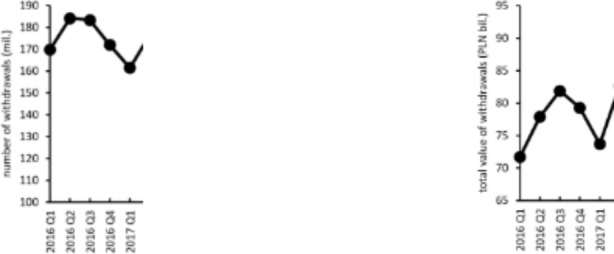
Total number of withdrawals (left panel) and the total value of withdrawals (right panel) from ATMs in Poland in the period 2016–2020

To provide a suitable background for the analysis of the impact of the COVID-19 pandemic on the behavior of ATM customers, in Table 1 we present quarterly data sourced by the Polish Central Bank (NBP) regarding the number and size of withdrawals from all ATMs located in Poland. These data are also presented in Fig. 2. As was mentioned in the previous section, the role of cash has changed in recent years and continues to do so. These changes are also visible in the data presented. The total number of withdrawals shows a downward trend, whereas the total value of withdrawals and the average value of a single withdrawal are characterized by upward trends.

The last four values of each variable relate to the time of the COVID-19 pandemic. Comparing them with the values from the previous years, our attention is drawn to the clear impact of the pandemic on the values of the variables describing ATM withdrawals. In particular, it is noticeable that the total number of withdrawals in the subsequent quarters of 2020 is much lower than in the corresponding periods of the previous years (even taking into account the trend observed in the data). The strongest changes are noticeable in the first and fourth quarters of 2020. In the first quarter, the COVID-19 pandemic began in Poland, and at that time the first restrictions were also introduced. Moreover, this was a period of great uncertainty regarding the nature of SARS-CoV-2 and the development of the pandemic. In the next few months, after this initial period and very significant restrictions, the pandemic situation in Poland improved, which led to the lifting of some of the restrictions. However, at the end of the year (in the fourth quarter), some restrictions were introduced again.

**Table 1:** Total number of withdrawals, total value of withdrawals, and average value of a single withdrawal from ATMs in Poland in the period 2016–2020

	Quarter of the year	2016	2017	2018	2019	2020
Total number of withdrawals (in millions)	1	169.813	161.415	158.286	149.080	133.425
	2	184.087	176.535	172.710	163.361	112.078
	3	183.303	174.202	167.493	162.306	136.504
	4	171.907	167.432	158.612	155.019	118.794
Total value of withdrawals (PLN billion)	1	71.686	73.683	76.296	78.943	83.168
	2	77.863	82.626	85.102	88.104	75.678
	3	81.878	85.357	86.929	90.615	85.369
	4	79.268	83.519	84.898	88.885	81.387
Average value of a single withdrawal (PLN)	1	422.15	456.48	482.01	529.53	623.33
	2	422.97	468.05	492.75	539.32	675.23
	3	446.68	489.99	519.00	558.30	625.39
	4	461.11	498.82	535.25	573.38	685.11

The total value of withdrawals in the initial phase of the pandemic, i.e. in the first quarter of 2020, continued the trend set by previous years. This fact, together with the reduced number of withdrawals, suggests that given the increasing uncertainty about the development of the pandemic, many people chose to withdraw cash rather than keep their savings in banks. As early as the second quarter there was a large decline in the total value of ATM withdrawals, accompanied by an even stronger drop in the number of withdrawals from ATMs. This may be the result of an earlier accumulation of cash reserves, the restrictions that had been introduced and the fact that during this period more and more people started making cashless payments. These changes are reflected in the average value ​​of a single withdrawal in each quarter. The average value of a withdrawal in the periods of the strictest pandemic restrictions, i.e. in the second and fourth quarters of 2020, is the highest of the entire period presented in Table 1.

The main part of the analysis presented in this paper is based on daily data on withdrawals from a sample of 81 ATMs belonging to the one of the largest ATM networks in Poland. All the ATMs under consideration are located in Krakow, which is the second most populous city in Poland. For each ATM we take into account the daily total value of withdrawals, the daily number of withdrawals, and the daily average value of a single withdrawal, defined as the quotient of the two previous quantities. Both the total value and the average value are expressed in PLN. The data used in the study cover the period January 1, 2018 – March 31, 2021.

The long-term tendencies observed in the aggregated data presented above are also visible in the data for individual ATMs. Therefore, a linear trend was removed from each of the time series of values describing withdrawals from the individual ATMs under investigation. In each case, the parameters of the trend were estimated on the basis of data up until the end of 2019.

In order to examine the impact of the COVID-19 pandemic on the behavior of ATM customers, described by the number of withdrawals or the value of a withdrawal, we employ event study analysis. This method was first designed to investigate how some events (such as announcements of earnings, or mergers and acquisitions) impact stock prices (Ball and Brown 1968; MacKinlay 1997; McWilliams and Siegel 1997), but it was later applied to various types of data, such as trading volume, volatility or transactional data (Murg et al. 2016; Gurgul and Majdosz 2007; Gurgul and Wójtowicz 2014, 2020; Suliga and Wójtowicz 2019; Gurgul and Suliga 2020). It is also helpful in examining market efficiency (Dragota and Tilica 2014).

In brief, event study analysis allows us to verify whether the behavior of a given time series observed at the time of an event (just before it or immediately after it) deviates from its normal behavior, which would have been observed if the event had not occurred. The significance of the impact of an event can be tested not only at the moment of its occurrence, but also throughout the entire event window, i.e. in the period around the event. In this paper, we examine the impact of various COVID-19 pandemic restrictions on changes in how ATMs are used. In particular, we analyze their impact on the daily total value of withdrawals, the daily number of withdrawals, and the daily average value of a single withdrawal. For $i$-th ATM, let us denote by${X}_{it}$ the value of a variable we are interested in on day $t$, for example the daily number of withdrawals. The study of the significance of the impact of a given event consists in verifying the significance of abnormal values of ${X}_{t}$, denoted by ${AX}_{it}$the difference between the observed values ${X}_{t}$ and their expected values $E\left({X}_{it}|{\Omega }\right)$ estimated on the basis of the data from an estimation window:

1 $${AX}_{it}={X}_{it}-E\left({X}_{it}|{\Omega }\right).$$

When ${X}_{t}$ is the daily number of withdrawals, its abnormal value ${AX}_{it}$ describes an additional change in the daily number of withdrawals implied by the event when compared to the number of withdrawals observed in the estimation window. If the mean of the abnormal number of withdrawals ${AX}_{it}$ at a given time $t$ in the event window is significantly greater than zero, this indicates that as a result of the event the daily number of withdrawals increased significantly above their normal level determined on the basis of data from the estimation window. Similarly, $A{X}_{it}$ significantly lower than zero indicates a negative impact of the event and a drop in the number of withdrawals below the normal level measured in the estimation window.

Many events related to the COVID-19 pandemic may be considered important in the context of the analysis presented in this paper. In order to investigate the effect of restrictions on the variables describing ATM withdrawals, the closure of schools and universities on March 12, 2020 was chosen as the main event. However, to take into account other important events (and to analyze changes in the behavior of ATM customers over a longer period), the event window in which the significance of changes in ATM withdrawal variables is examined covers the period from February 1, 2020 to March 31, 2021. If March 12, 2020 is denoted by $t=0$, then the event window includes daily data for $t = -40, ..., 384$. As described below, the Kolari-Pynnönen (2011) test allows us to verify the significance of ${AX}_{it}$ for each $t$ in the event window separately. Hence, the choice of the main event (i.e. $t=0$) does not impact the results of the event study analysis presented in this paper. The most important element is the choice of the event and the pre-event window.

Taking the possible high volatility of daily data into account, the analysis is also based on weekly data on the characteristics of ATM withdrawals. In this case, $t=0$ denotes the week beginning March 12, 2020, i.e. from March 12 (Thursday) to March 18 (Wednesday). This definition also determines how the daily to weekly data are aggregated. The data for each week are calculated on the basis of daily data from Thursday to Wednesday. Given this definition of the event and data under consideration, the event window contains weekly data from February 13, 2020 (Thursday) to March 31, 2021 (Wednesday), i.e. for $t=-4, \dots , 54$. Regardless of whether daily or weekly data are tested, the estimation window covers all data from 2019. In the case of daily data, we denote the estimation window as $t=-436, \dots ,-72$, whereas for weekly data as $t=-62, \dots ,-11$. This definition of the estimation window ensures that it contains enough data to estimate the normal behavior of the variable undergoing analysis correctly, and that the estimation procedure for the normal behavior of these variables is not affected by any pandemic-related events. This also allows us to compare the level of ATM withdrawal during the pandemic with the level from the period before it.

Various methods of estimating $E\left({X}_{it}|{\Omega }\right)$ can be found in the literature, although in this paper, to compute the expected value of a variable for weekly data, we employ a constant mean model in which $E\left({X}_{it}|{\Omega }\right)$ has the same value as the average of ${X}_{t}$ from the estimation window. This method of estimating $E\left({X}_{it}|{\Omega }\right)$ is simple, but very useful and robust. In order to take the weekly seasonality of withdrawals into account (Gurgul and Suder 2016), in the analysis of daily data we estimate $E\left({X}_{it}|{\Omega }\right)$ separately for each day of a week.

In this paper, the event study method is used not to analyze the impact of the pandemic on the number of withdrawals from a single ATM, but to study its impact on all the ATMs under consideration. To verify the significance of the expected values of abnormal ${X}_{it}$ in the event window, Kolari and Pynnönen’s (2011) generalized rank test is employed. The procedure for this test is presented in detail in Gurgul and Wójtowicz (2014, 2020). The great advantage of it is that it does not require any assumption about the normality of data. Additionally, it is able to cope with the existence of possible cross-correlation of data from different ATMs.

We describe the test procedure, using our analysis of daily data on the number of withdrawals from ATMs as an example. The test statistics are constructed as follows. For each $i$-th ATM in the dataset ($i=1,\dots ,N$), for $t = -438, ...,-72,-40,\dots , 384$ we compute the abnormal number of withdrawals ${AX}_{it}$ from (1) with $E\left({X}_{it}|{\Omega }\right)$, calculated previously as the average daily number of withdrawals in the estimation window (i.e. in 2019). Then, all abnormal numbers of withdrawals in the event and estimation windows are standardized:

2 $${SAX}_{it}={AX}_{it}/{S}_{{AX}_{i}},$$

where ${S}_{{AX}_{i}}$ is the standard deviation of ${AX}_{it}$ in the estimation window. This procedure ensures the comparability of the abnormal number of withdrawals computed on the basis of data from different ATMs.

To investigate the influence of the pandemic restrictions we test the significance of the abnormal number of withdrawals for each ${t}_{0}$ in the event window separately. Therefore, for each ${t}_{0}=-40,\dots ,384$ the demeaned standardized abnormal ranks of ${AX}_{it}$s are given by the formula:

3 $${U}_{it}=\frac{rank\left({AX}_{it}\right)}{T+1}-1/2$$

for $i=1,\dots ,N$, where $t\in {\Theta }=\{-438,\dots ,-72,{t}_{0}\}$, $T-1$ is the length of the estimation window and $rank\left({AX}_{it}\right)$ denotes the rank of ${AX}_{it}$ within the vector that consists of the abnormal number of withdrawals from the estimation window and ${AX}_{i{t}_{0}}$. In this notation ${U}_{i{t}_{0}}$ refers to the demeaned standardized abnormal rank of ${AX}_{i{t}_{0}}$ and the null hypothesis of no event effect is equivalent to

4 $$E\left({U}_{i{t}_{0}}\right)=0.$$

To verify this hypothesis, we employ the generalized rank ${t}_{grank}$ test statistic of Kolari-Pynnönen (2011) defined as:

5 $${t}_{grank}=Z\sqrt{\frac{T-2}{T-1-{Z}^{2}}} ,$$

where $$Z={\overline{U}}_{{t}_{0}}/{S}_{\overline{U}}$$, $${S}_{\overline{U}}=\sqrt{\frac{1}{T}\sum _{t\in {\Theta }}{\overline{U}}_{t}^{2}}$$ and $${\overline{U}}_{t}=\frac{1}{N}\sum _{i=1}^{N}{U}_{it}$$.

Under the null hypothesis of no effect,﻿﻿ the distribution of ${t}_{grank}$ statistic converges to Student *t* distribution with $T-2$ degrees of freedom when the number of ATMs $N$ increases.

A natural measure of the impact of the event on a variable under investigation (for example on the daily number of withdrawals) is the average ${\widehat{AX}}_{t}$ of the abnormal values of ${X}_{it}$:

6 $${\overline{AX}}_{t}=\frac{1}{N}\sum _{i=1}^{N}{AX}_{it}.$$

The average ${\overline{AX}}_{t}$ measures how much higher (or lower) the values of ${X}_{t}$ at time $t$ are when compared to the data from the estimation window.

## Results

### Weekly data

We begin our empirical analysis of the changes in the behavior of ATM customers caused by the COVID-19 pandemic with a study of the weekly data described in the previous section. Analyzing data aggregated in this way will allow us to assess roughly when significant changes in the value and number of ATM withdrawals occurred. As it is obvious that the greatest changes in customer habits took place at the very beginning of the pandemic, or just before the official announcement of the restrictions, detailed results of the analysis are presented for the first sixteen weeks of the event window (for $t=-4,\dots ,11$) covering the period from February 13 up to June 3. Table 2 reports the average abnormal number of withdrawals (${\overline{AN}}_{t}$), the average abnormal total value of withdrawals (${\overline{ATV}}_{t}$), and the average abnormal value of a single withdrawal (${\overline{ASV}}_{t}$). Together with these averages we report the values of ${t}_{grank}$ statistics from the Kolari-Pynnönen significance test.

One of the most visible things in Table 2 is the negative averages of the abnormal daily number of withdrawals ${\overline{AN}}_{t}$. They indicate that in the period under consideration the average daily number of withdrawals from a single ATM was lower than in the pre-pandemic period (i.e. in 2019). This is in line with the results for all ATMs in Poland presented in Table 1; Fig. 2. However, the results of the event study analysis in Table 2 give additional information. Some of the ${\overline{AN}}_{t}$s, at the beginning of the event window, are insignificant. This indicates that the activity of ATM customers in February 2020 did not differ from their activity in the previous year. A significantly negative change in the daily number of withdrawals first appears for $t=0$, i.e., between March 12 and March 18. This was right after the authorities in Poland announced (on March 11) the closure of schools and universities and the suspension of museums and other cultural institutions from March 12. This was the very beginning of pandemic restrictions in Poland. The value of the average ${\overline{AN}}_{0}$ means that on average the number of withdrawals from each ATM dropped by about 25 per day in the first week of the pandemic restrictions when compared to the pre-pandemic period in 2019. In the following weeks, further restrictions were imposed leading to even greater (and significant) drops in the number of ATM withdrawals. After about 10 weeks of the pandemic, at the beginning of June, negative changes in customers’ activity diminished (and were significant only at a level of 10%). In subsequent weeks the averages ${\overline{AN}}_{t}$ (not displayed in Table 2) were still negative, but, in majority of cases, they were insignificant. However, from mid-October to mid-February 2021, the averages ${\overline{AN}}_{t}$ were again significantly negative (at a level of 5%), indicating the second period of significantly reduced ATM customer activity. It is worth noting that at the beginning of October various pandemic restrictions were introduced once again.

.

**Table 2:** Results of the event study analysis for weekly data

	Total value of withdrawals (in thousands PLN)		Number of withdrawals		Value of a single withdrawal (in PLN)	
$t$	${\overline{ATV}}_{t}$	${t}_{grank}$	${\overline{AN}}_{t}$	${t}_{grank}$	${\overline{ASV}}_{t}$	${t}_{grank}$
-4	-0.87	-0.41	-3.14	-0.53	1.54	-0.01
-3	-2.55	-0.92	-8.30	-1.19	5.47	0.09
-2	0.88	0.51	-3.68	-0.39	39.85^*^	1.86
-1	4.67	1.36	0.38	0.22	57.48^***^	2.68
0	5.63	0.92	-25.15^**^	-2.04	271.99^***^	3.86
1	-11.74^**^	-2.35	-42.89^**^	-2.62	82.04^***^	2.90
2	-12.02^**^	-2.50	-43.35^**^	-2.64	76.00^***^	2.70
3	-10.20^**^	-2.21	-39.84^**^	-2.55	79.22^***^	2.95
4	-11.16^**^	-2.40	-40.55^**^	-2.59	52.89^**^	2.18
5	-11.48^**^	-2.42	-39.13^**^	-2.56	62.40^**^	2.53
6	-10.45^**^	-2.33	-35.39^**^	-2.43	42.17^**^	2.03
7	-10.64^**^	-2.33	-37.00^**^	-2.51	36.49^**^	2.04
8	-6.44	-1.51	-27.41^**^	-2.12	36.36^**^	2.34
9	-9.10^**^	-2.15	-28.02^**^	-2.24	-4.95	0.52
10	-9.28^**^	-2.21	-25.52^**^	-2.19	-27.77	-0.62
11	-6.37	-1.53	-20.23^*^	-1.76	10.81	0.48

Notes: ^*^, ^**^, ^***^ indicate the significance of a mean at 10%, 5% and 1%, respectively, which results from from Kolari-Pynnönen’s rank test

**Fig. 3:**
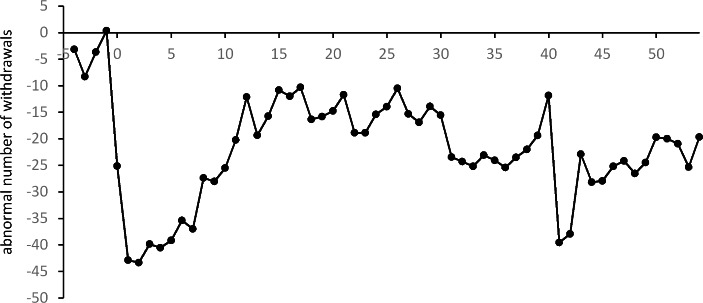
Averages ${\overline{AN}}_{t}$of abnormal daily number of ATM withdrawals in subsequent weeks of the event window

The above analysis of ${\overline{AN}}_{t}$ values shows that the number of withdrawals throughout the pandemic period (from March 2020 and in 2021) was lower than in the pre-pandemic period. As an illustration of these changes in customers’ behavior, in Fig. 3 we present the values of the averages ${\overline{AN}}_{t}$ for each week in the whole event window up to the end of March 2021. For most of this period the number of withdrawals was significantly lower than in 2019 (at least at a level of 10%). In general, ${\overline{AN}}_{t}$ is insignificant only between June and October 2020 (for $t=12,\dots ,26$). This coincides with the period of relative calm, which lasted a few months, after which some of the restrictions were restored at the beginning of October. In Fig. 3, we notice a large decrease in the number of withdrawals after $t=40$. However, this sudden and significant change may have been caused by Christmas and the New Year holidays rather than the pandemic.

**Fig. 4:**
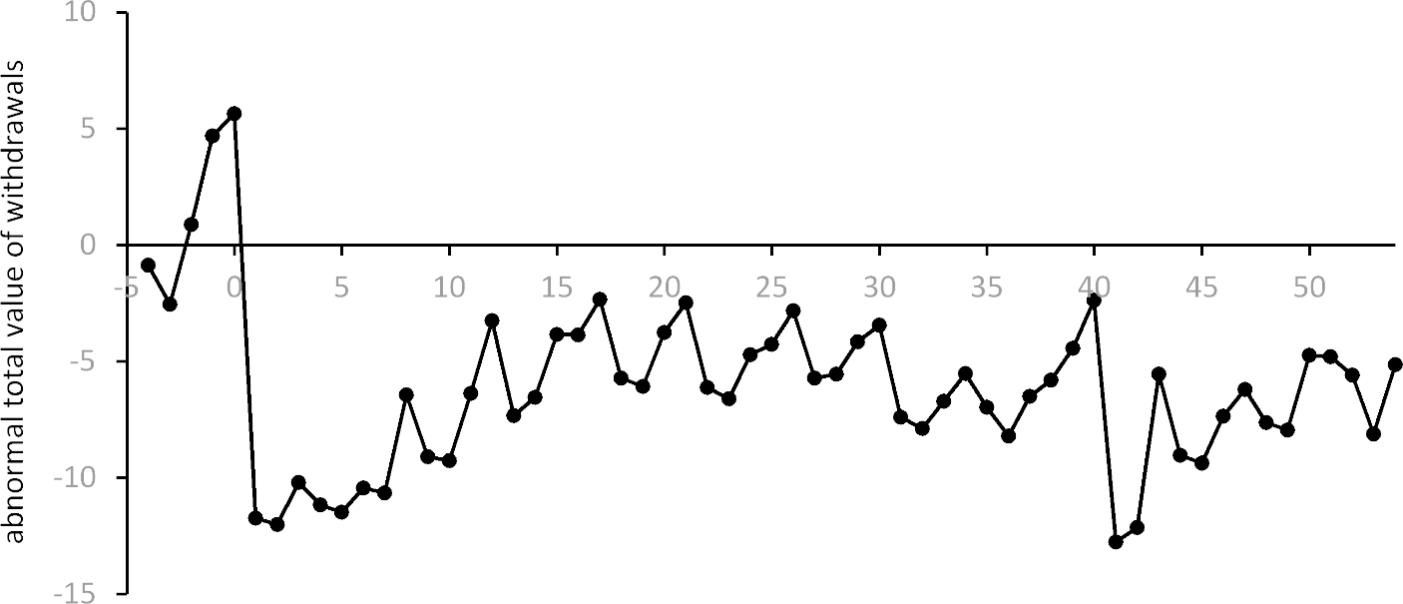
Averages ${\overline{ATV}}_{t}$ of abnormal daily total value of ATM withdrawals in subsequent weeks of the event window

**Fig. 5:**
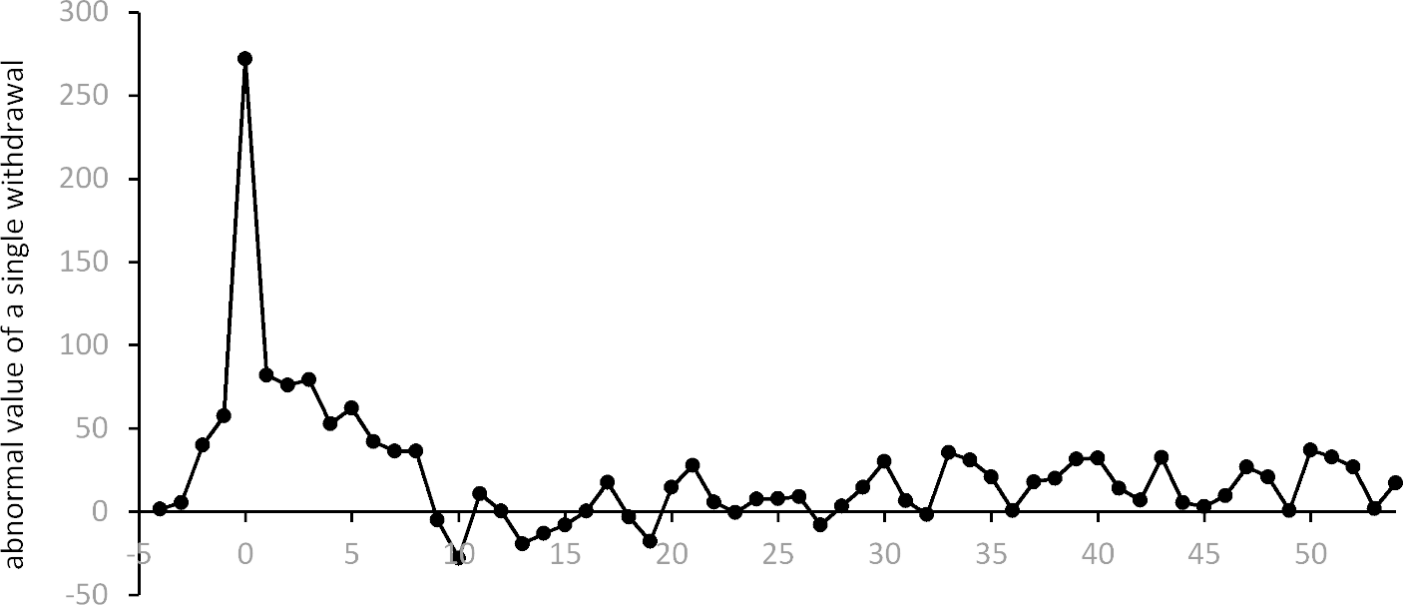
Averages ${\overline{ASV}}_{t}$ of abnormal value of a single withdrawal in subsequent weeks of the event window

The COVID-19 pandemic also resulted in a significant decrease in the total daily value of ATM withdrawals. Significantly negative ${\overline{ATV}}_{t}$ is observed for $t=1,\dots ,7$ and $t=\text{9, 10}$. The positive values of the abnormal average daily total value of withdrawals observed just before the introduction of the pandemic restrictions (and even in the first week when schools and universities were closed) are insignificant when compared to the values from 2019. This means that at the beginning of the pandemic the total value of withdrawals was greater than in 2019, despite the smaller number of withdrawals. A comparison of the results for the total value and the number of withdrawals shows that a significant drop in the total value of withdrawals was observed one week later than a significant drop in the number of withdrawals. However, for both of these characteristics of customers’ activity, the negative effect started to diminish after about 10 weeks, at the beginning of June. The values of the averages ${\overline{ATV}}_{t}$ presented in Fig. 5 show a similar pattern to the averages ${\overline{AN}}_{t}$ in Fig. 4. In fact, they are highly correlated (with Pearson correlation equal to 0.94). Despite this strong relationship, the values of the averages ${\overline{ATV}}_{t}$ are significantly negative in many fewer cases than the averages of the daily number of withdrawals. They were only significant at a level of 5% in January 2021.

The differences in the changes in the number of ATM withdrawals and the total value of these withdrawals can be explained by changes in the size of a single withdrawal. A significant increase in the value of a single withdrawal is noticeable at the very beginning of the period. As can be seen in the two last columns of Table 2, the average abnormal value for a single withdrawal is significantly positive (at a level of 10%) for $t=-2$ (between February 27 and March 4), i.e. two weeks before the first significant ${\overline{AN}}_{t}$ and three weeks before the first significant ${\overline{ATV}}_{t}$. The highest value of a single withdrawal is observed for $t=0$ (between March 12 and March 18) when, on average, users withdraw about PLN 272 more in one ATM withdrawal when compared to the average value of a single withdrawal in 2019. A comparison of the moments of significant changes in the number of withdrawals, the size of a single withdrawal and the total value of withdrawals indicates that ATM users started stockpiling cash a few weeks before formal restrictions were introduced (based on rumors and their own predictions). Initially, they increased the amount of cash withdrawn only slightly. In the following weeks, when the introduction of restrictions became more and more certain, the size of individual withdrawals increased sharply. At the same time, withdrawals became less frequent. As a result, the total withdrawal value remained unchanged up to t = 1. As can also be seen in Fig. 5, the value of a single withdrawal returned relatively quickly to the pre-pandemic level. From $t=9$ (a week between May 14 and May 20), the means of the abnormal values of a single withdrawal are insignificant. For the rest of the event window, significant abnormal values of a single withdrawal appear incidentally, without much connection to the restrictions introduced. An analysis of Table 2; Fig. 5 indicates that the behavior of ATM customers (reflected in the positive abnormal values of a single withdrawal) started to change significantly a few weeks before formal pandemic restrictions were introduced in Poland. This increase in the demand for cash at a time of great uncertainty should be taken into account by managers of ATM networks.

### Daily data

To give a more detailed picture of changes in ATM withdrawals at the beginning of the pandemic in Poland, we repeated the above analysis, applying it to daily data. Table 3 reports the results for the most interesting time at the beginning of March 2020, when the first pandemic restrictions were imposed and significant changes in weekly data on withdrawals appeared for the first time.

**Table 3:** Results of the event study analysis for daily data

		Total value of withdrawals (in thousands PLN)		Number of withdrawals		Value of a single withdrawal (in PLN)	
Date	$t$	${\overline{ATV}}_{t}$	${t}_{grank}$	${\overline{AN}}_{t}$	${t}_{grank}$	${\overline{ASV}}_{t}$	${t}_{grank}$
2020-03-08	-4	0.24	0.29	2.93	0.28	-9.96	-0.37
2020-03-09	-3	5.66	1.27	-0.56	0.10	73.86^***^	2.81
2020-03-10	-2	12.49^**^	2.22	8.72	1.09	102.17^***^	3.90
2020-03-11	-1	12.96^*^	1.93	3.14	0.37	143.41^***^	4.15
2020-03-12	0	12.24^*^	1.90	-5.81	-0.34	224.63^***^	5.07
2020-03-13	1	29.53^**^	2.13	-7.00	-0.08	346.25^***^	5.46
2020-03-14	2	14.91	1.33	-17.59	-0.86	372.38^***^	5.81
2020-03-15	3	5.03	0.71	-21.58	-1.93	427.02^***^	4.71
2020-03-16	4	-4.55	-1.14	-43.02^*^	-2.64	223.44^***^	3.36
2020-03-17	5	-7.47^*^	-1.65	-45.76^***^	-2.93	238.65^***^	4.03
2020-03-18	6	-11.21^**^	-2.26	-42.94^***^	-2.89	70.83^**^	2.46
2020-03-19	7	-11.56^**^	-2.32	-44.31^***^	-2.81	76.02^***^	3.32
2020-03-20	8	-16.12^***^	-2.91	-54.41^***^	-3.02	74.34^**^	2.14
2020-03-21	9	-10.15^**^	-2.37	-43.60^***^	-2.90	89.22^**^	2.11
2020-03-22	10	-5.63	-1.24	-24.98^**^	-2.30	124.70^***^	2.87
2020-03-23	11	-13.72^***^	-2.91	-45.95^***^	-3.00	65.18^*^	1.68
2020-03-24	12	-10.06^**^	-2.30	-41.45^***^	-2.81	67.69^**^	2.50
2020-03-25	13	-11.83^***^	-2.62	-45.89^***^	-3.01	76.38^***^	2.87

On the basis of the results from Table 3 we can distinguish several days that were significant in terms of changes in ATM customers’ behavior. The first of them is March 9 – the day before some important restrictions were announced (for example, the cancelation of mass events). This was the first day when we observe a significantly positive (at a level of 1%) abnormal value of a single withdrawal, indicating a significantly higher (than in 2019) value of a single withdrawal. March 15 (Sunday), the day before further, more serious, restrictions were announced, was the beginning of the period of significantly positive values of ${\overline{ASV}}_{t}.$On this day, the average value of a single ATM withdrawal was higher by about PLN 427 than in the pre-pandemic period. From that day on, the average value of payouts decreased (see Fig. 8), and significant abnormal values of single withdrawals continued to be noticeable, in general, until mid-May 2020 ($t=61$).

**Fig. 6:**
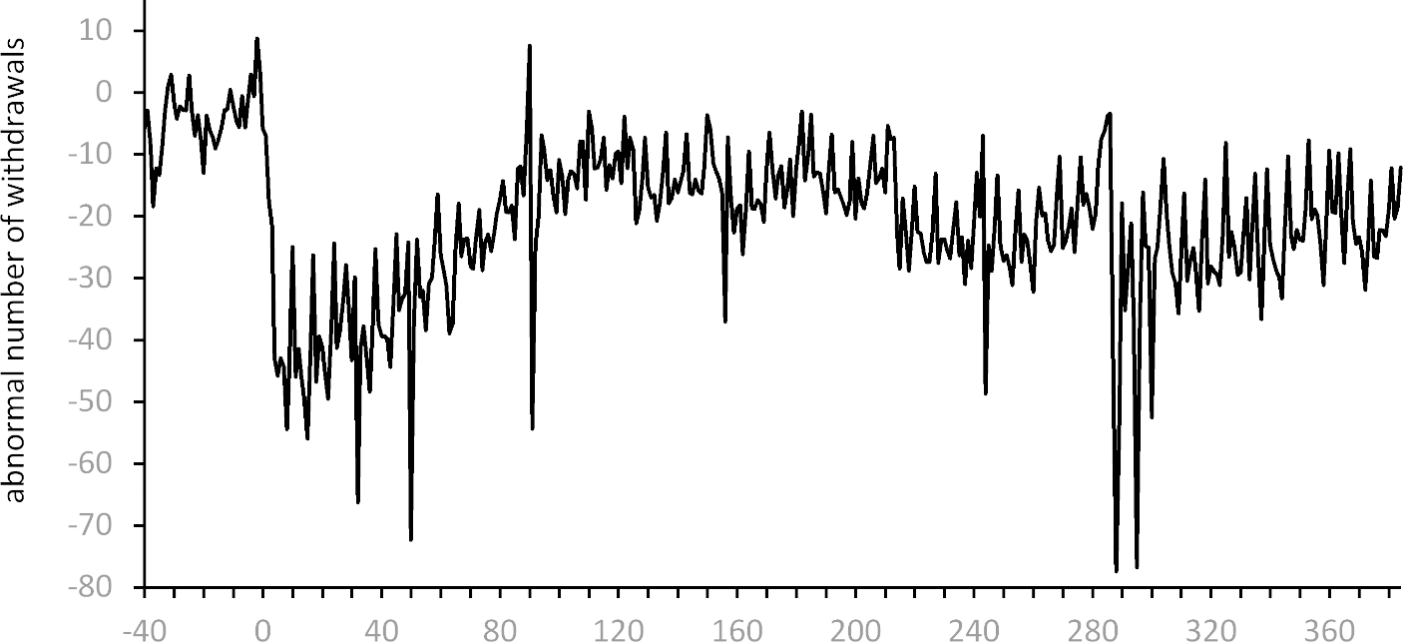
Averages ${\overline{AN}}_{t}$ of the abnormal daily number of ATM withdrawals on subsequent days of the event window

Sunday, March 15, was also the first day when the number of withdrawals was significantly lower than in 2019. Additionally this was also the beginning of the very long period when abnormal numbers of withdrawals had significantly negative values (see Fig. 6). In general, a significantly lower number of daily ATM withdrawals is observable until the end of the period in question. These negative values of ${\overline{AN}}_{t}$ are in line with the pattern observed in Table 1; Fig. 2. However, in the context of cash supply management it is important to notice that after a very dramatic drop in the number of withdrawals at the beginning of March, an upward trend is apparent. After about three months, at the beginning of June, the values of ${\overline{AN}}_{t}$ stabilize at a new level.

Despite the lower number of withdrawals at the end of February and at the beginning of March, positive abnormal total values of withdrawals are noticeable. Significantly positive ${\overline{ATV}}_{t}s$ occured between March 10 and March 13 (i.e. just after the announcements of the first restrictions) with the highest value being around 29.5 (on Friday, March 13). This value indicates that on that day the total value of withdrawals from a single ATM was around PLN 29 500 higher than in 2019. A few days later, the total values of withdrawals dramatically decreased, resulting in significantly negative averages ${\overline{ATV}}_{t}$. From Fig. 6 we notice that from that time values of ${\overline{ATV}}_{t}$ remained at a similar level up to the end of the event window.

It is worth noting that positive abnormal averages of a single withdrawal and significantly positive abnormal total values of withdrawals can be seen from the end of February 2020 in Fig. 8. For example, ${\overline{ASV}}_{t}$ on February 29 is about PLN 51 while ${\overline{ATV}}_{t}$ is about 1.7. These positive values indicate that the increased demand for cash appeared much earlier than the pandemic restrictions were introduced. This also suggests that due to the growing uncertainty about the future situation in Poland, ATM customers started creating a reserve of cash about two weeks before strict pandemic restrictions were announced and introduced. This observation is challenging in the context of the management of the ATM network. The formal introduction of restrictions only resulted in a reduction in the number of withdrawals. This also led to a significant decrease in the total sum of withdrawals a few days later. The introduction of further restrictions did not have a significant impact on changes in ATM customers’ behavior. The gradual lifting of the restrictions (announced mostly on May 30) resulted in the stabilization of the total value of daily ATM withdrawals, the number of withdrawals as well as the value of a single withdrawal at new levels that were close to the levels observed before the pandemic period. It is particularly significant that these positive changes were triggered once again by the announcement of plans to lift the restrictions, not by the lifting of the restrictions themselves.

**Fig. 7:**
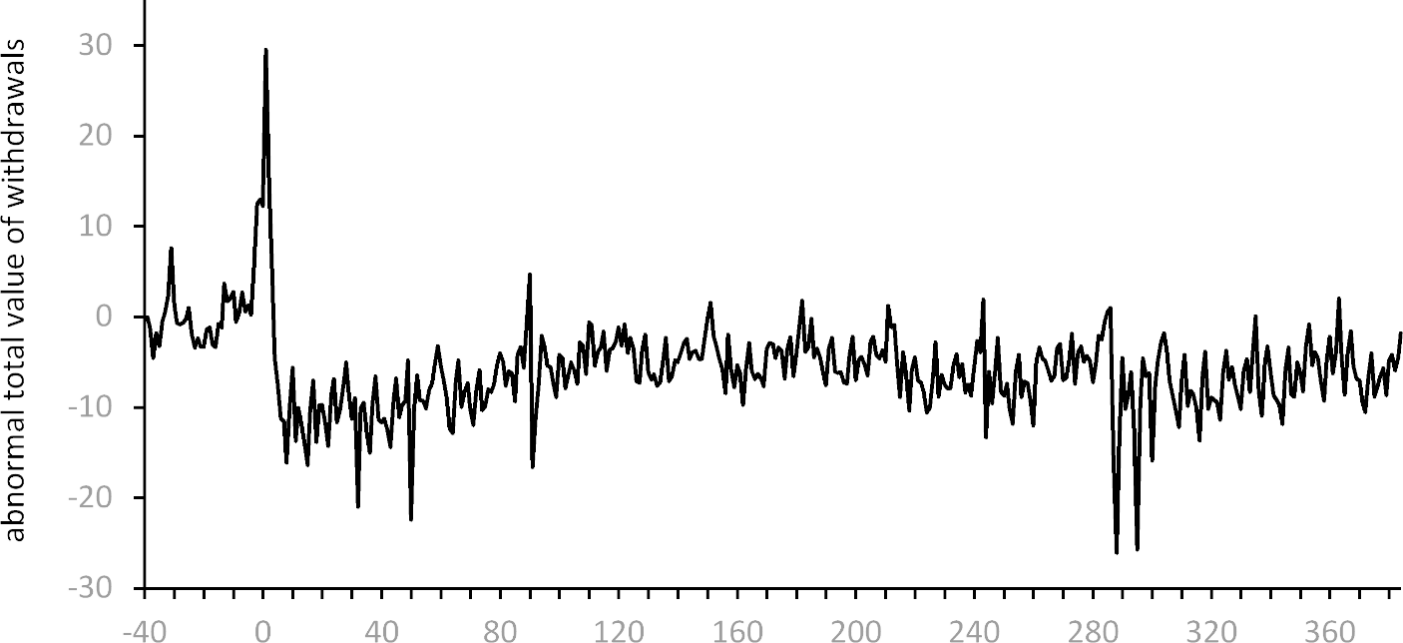
Averages ${\overline{ATV}}_{t}$ of the abnormal daily total log-value of ATM withdrawals on subsequent days of the event window

**Fig. 8:**
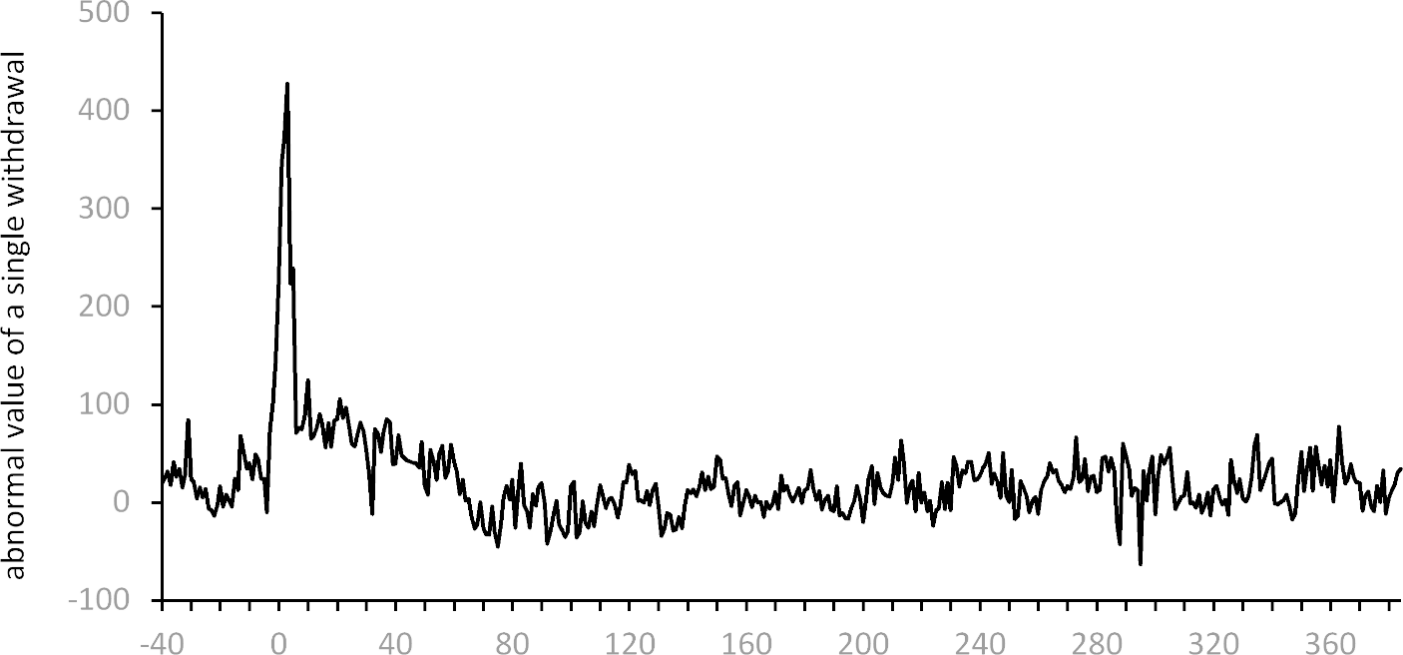
Averages ${\overline{ASV}}_{t}$ of the abnormal value of a single withdrawal on subsequent days of the event window

Different behavior could be observed during the second wave of restrictions, which were introduced in October 2020. At that time there was a significant drop in the number of ATM withdrawals. However, the value of a single withdrawal did not differ significantly from the value in 2019. This means that, unlike the first wave, ATM customers were not hoarding cash. The experiences of the previous few months changed their approach, as it showed that the restrictions that had been introduced would not cause difficulties related to access to money.

## Conclusion

In this paper we have analyzed the impact of the COVID-19 pandemic on the behavior of ATM customers in Poland on the basis of 81 time series of data on withdrawals from ATMs in Krakow. Event study analysis allowed us to determine very precisely the days on which there were significant changes in the value of ATM withdrawals, the number of withdrawals, as well as the average value of a single withdrawal. To a large extent, the results of the study presented in this paper confirm the results of other research related to the impact of the COVID-19 pandemic on ATM operations (e.g. Alharthi et al. 2021). In particular, our results confirm that, in general, a pandemic leads to a decrease in demand for cash (Cevik 2020). The total value of ATM withdrawals shifted during the pandemic period to a level significantly lower than previously. However, the most interesting observations concern the beginning phase of the pandemic, when there was great uncertainty surrounding the development of the pandemic and the actions of the government. In this turbulent time (about one week before the pandemic restrictions were introduced and a few days after them), the total daily value of withdrawals increased significantly. This was a sign of ATM customers hoarding cash. However, the restrictions introduced significantly reduced the number of withdrawals and halted the increase in the total value of withdrawals. Since then, the total value of withdrawals has remained at a lower level.

The sudden changes in ATM customer behavior described in this paper present the managers of the ATM network with a serious challenge, because forecasting is very difficult for them as they have to anticipate the government’s actions and the restrictions introduced. The only indication of the possibility of changes in the demand for cash is increased uncertainty. The significance of the impact of uncertainty on changes in this demand is demonstrated by the behavior of ATM users at the beginning of the second wave of the pandemic at the end of 2020. At that time, some of the restrictions were re-introduced. However, since users had experience from the previous months of the pandemic, this did not result in such large changes in the daily values of withdrawals or in the number of withdrawals as at the very beginning of the pandemic.

The results of this article imply that rumors may be more important than official regulations or restrictions when managing an ATM network. This is because the behavior of society is very often influenced by rumors. The results also show how great a sudden change in the demand for cash can be. In the future, in the face of an emerging crisis, this will make it possible to adjust forecasts accordingly.

The observation that, in the short term, the main role in the change in ATM customer behavior is played by uncertainty rather than restrictions introduced, prompts the conclusion that the results of the study described in this paper can be generalized to other crises. However, it should be noted here that changes in the behavior of ATM customers in the face of a crisis can depend on various factors. Among them is, of course, the nature and extent of the crisis. Nevertheless, people’s trust in the authorities and in the government, for example, is also important (the higher it is, the calmer the expected reaction). Cultural, and even historical factors can also play a significant role.

Obviously, the conclusions of this article have their limitations. They result, inter alia, from the fact that the research was carried out on the basis of data from only one city. Moreover, the data concerned only one ATM network. On the other hand, the authors’ experience shows that extending the sample would give similar results and conclusions. Conducting a survey among ATM users would further define which events (and how events) affect changes in their behavior regarding the demand for cash. A more detailed study would also investigate changes in user behavior depending on the location of the ATM (see, for example, Gurgul and Suder 2014).

## Footnotes

1 It is worth mentioning that at some universities classes and lectures were canceled on March 11; information about this had been announced on March 10.
